# Supplementary material for: Gene and protein expression of mTOR and LC3 in hepatocellular carcinoma, colorectal liver metastasis and “normal” liver tissues
Source: PLoS One. 2020 Dec 23;15(12):e0244356. doi: 10.1371/journal.pone.0244356 (PMC7757890; doi:10.1371/journal.pone.0244356)
Supplement: S1 Fig — (DOCX) [file pone.0244356.s001.docx]

original raw image files <https://data.mendeley.com/datasets/kg2rvwnprh/draft?a=eca9af40-018a-4200-9176-50a889bf5898>
