# Supplementary material for: Gene and protein expression of mTOR and LC3 in hepatocellular carcinoma, colorectal liver metastasis and “normal” liver tissues
Source: PLoS One. 2020 Dec 23;15(12):e0244356. doi: 10.1371/journal.pone.0244356 (PMC7757890; doi:10.1371/journal.pone.0244356)
Supplement: S2 Table — (PDF) [file pone.0244356.s003.pdf]

ID	gender	age	pathology	virus	hepatitis	geno
1	0		1	2	1	1
2	1	52	1	2	1	1
3	0	36	1	2	1	1
4	0	21	1	2	1	1
5	1	29	1	2	1	1
6	1	39	1	2	1	1
7	0	44	1	2	1	1
8	0	32	1	2	1	1
9	0	53	1	2	1	1
10	1	50	1	2	1	1
11	1	46	1	2	1	1
12	1	35	1	2	1	1
13	0	54	1	2	1	1
14	1	67	1	2	1	1
15	1	43	1	2	1	1
16	0	55	1	2	1	1
17	1	38	1	2	1	1
18	0	41	1	2	1	1
19	1	36	1	2	1	2
20	1	55	1	2	1	2
21	1		1	2	1	2
22	1	57	1	2	1	2
23	1	49	1	2	1	2
24	0	21	1	2	1	2
25	0	58	1	2	1	2
26	1	56	1	2	1	2
27	0	57	1	2	1	2
28	1	27	1	2	1	4
29	1	34	1	2	1	4
30	1	36	1	2	1	4
31	1	20	1	2	1	3
32	1	32	1	2	1	3
33	1		1	2	1	3
34	1	35	1	2	1	3
35	1	26	1	2	1	3
36	0	33	1	2	1	3
37	1	33	1	2	1	3
38	1	55	1	2	1	3
39	1	42	1	1	1	
40	1		1	1	1	
41	1	56	1	1	1	
42	1		1	1	1	
43	1	32	1	1	1	
44	0	47	1	1	1	
45	1	32	1	1	1	
46	1	28	1	1	1	
47	1	31	1	1	1	
48	1	31	1	1	1	
49	1	39	1	1	1	

**50**	1	29	1	1	1	
**51**	1	47	1	1	1	
**52**	1	42	1	1	1	
**53**	0	56	1	1	1	
**54**	1	60	1	1	1	
**55**	0	52	1	1	1	
**56**	1	40	1	1	1	
**57**	1	54	1	1	1	
**58**	1	51	1	1	1	
**59**	1	59	1	1	1	
**60**	0	34	1	1	1	
**61**	1	61	1	1	1	
**62**	1	53	1	1	1	
**63**	1	42	1	1	1	
**64**	1	61	1	1	1	
**65**	1	76	1	1	1	
**66**	1	55	1	1	1	
**67**	0	63	1	1	1	
**68**	1	69	1	1	1	
**69**	0	56	2	1	1	
**70**	1	47	2	1	1	
**71**	1	33	2	2	1	3
**72**	1	35	2	2	1	3
**73**	1	*42*	2	2	1	3
**74**	1		2	2	1	3
**75**	1	41	2	2	1	3
**76**	1	69	2	2	1	1
**77**	0	60	2	2	1	1
**78**	0	62	2	2	1	2
**79**	1	46	2	2	1	1
**80**	1	49	2	2	1	
**81**	1	60	2	2	1	
**82**	1	51	2	2	1	
**83**	0	60	3	2	1	1
**84**	1	66	3	2	1	1
**85**	1	60	3	2	1	1
**86**	1	63	3	2	1	2
**87**	0	69	3	2	1	1
**88**	0	75	3	2	1	1
**89**	1	23	3	2	1	1
**90**	1	71	3	2	1	1
**91**	0	78	3	2	1	1
**92**	0	72	3	?	1	
**93**	0	62	3	2	1	
**94**	0	71	3	2	1	
**95**	1	64	3	2	1	
**96**	0	69	3	2	1	
**97**	0	72	3	2	1	
**98**	1	83	3	2	1	
**99**	1	67	3	2	1	

**100**	1	78	3	2	1	
**101**	0	70	3	2	1	
**102**	1	60	3	2	1	
**103**	0	72	3	2	1	
**104**	1	77	3	2	1	
**105**	0	51	3	2	1	
**106**	1		3	2	1	
**107**	1	52	3	2	1	
**108**	1	66	3	2	1	
**109**	1	62	3	2	1	
**110**	1	66	3	2	1	
**111**	0	61	3	0	0	
**112**	1	65	3	0	0	
**113**	0	72	3	0	0	
**114**	1	46	3	0	0	
**115**	1	61	3	0	0	
**116**	1	58	3	0	0	
**117**	1	32	3	0	0	
**118**	1	64	3	0	0	
**119**	1	67	3	0	0	
**120**	1	51	3	0	0	
**121**	1	61	3	0	0	
**122**	1		3	0	0	
**123**	1	55	3	1	1	
**124**	1	78	3	1	1	
**125**	0	69	3	0	0	
**126**	1	43	3	1	1	
**127**	1	65	3	1	1	
**128**	0	66	3	1	1	
**129**	0	69	3	1	1	
**130**	1	27	3	1	1	
**131**	1	59	3	1	1	
**132**	1	52	3	1	1	
**133**		53	3	2	1	
**134**	1	66	3	1	1	
**135**	1	67	3	0	0	
**136**	0	50	3	0	0	
**137**	0	35	0	0	0	
**138**	1	61	3	0	0	
**139**	0	51	3	0	0	
**140**	0	67	3	0	0	
**141**	1	67	3	0	0	
**142**	1	67	3	0	0	
**143**	1	69	3	0	0	
**144**	1	62	3			
**145**	0	46	3	0	0	
**146**	1		3	0	0	
**147**	1	76	3	0	0	
**148**	1	60	3	0	0	
**149**	0		3	0	0	

**150**	1	63	3	0	0	
**151**	1	68	4	0	0	
**152**	1					
**153**	0	62	0	0	0	
**154**	1	47	0	0	0	
**155**	0	25	0	0	0	
**156**	0		0	0	0	
**157**	0	72	0	0	0	
**158**	0	31	0	0	0	
**159**	0	63	0	0	0	
**160**	0	47	4	0	0	
**161**	1	59	4	0	0	
**162**	0	58	4	0	0	
**163**	1	73	4	0	0	
**164**	0	69	4	0	0	
**165**	0	29	4	0	0	
**166**	1	70	4	0	0	
**167**	0	64	4	0	0	
**168**	1	61	4	0	0	
**169**	0	48	4	0	0	
**170**	1	65	4	0	0	
**171**	1	70	4	0	0	
**172**	1	73	4	0	0	
**173**	0	62	4	0	0	
**174**	0	61	4	0	0	
**175**	1	66	4	0	0	
**176**	0	40	4	0	0	
**177**	0	46	4	0	0	
**178**	1	60	4	0	0	
**179**	1	68	4	0	0	
**180**	1	64	4	0	0	
**181**	1	73	4	0	0	
**182**	1	66	4	0	0	
**183**	1	63	4	0	0	
**184**	1	55	4	0	0	
**185**	1	58	4	0	0	
**186**	0		4	0	0	
**187**	0	61	4	0	0	
**188**	1	62	4	0	0	
**189**	1	48	4	0	0	
**190**	1	77	4	0	0	
**191**	0	76	4	0	0	
**192**	1	51	4	0	0	
**193**	1	60	4	0	0	
**194**	1	54	4	0	0	
**195**	1	62	4	0	0	
**196**	0	56	4	0	0	
**197**	0	69	4	0	0	
**198**	1	67	4	0	0	
**199**	1	71	4	0	0	

**200**	0	74	4	0	0	
**201**	1	62	4	0	0	
**202**	0	76	4	0	0	
**203**	0	68	4	0	0	
**204**	0	54	4	0	0	
**205**	1	52	4	0	0	
**206**	0	56	4	0	0	
**207**	1	46	4	0	0	
**208**	1	61	4	0	0	

cause	chemo	mTOR_R_Nor	mTOR_R_Peri	mTOR_R_Tum
0				
0				
0		21.345		
0				
0		37.669		
0		57.013		
0		65.348		
0		39.132		
0		93.109		
0		62.124		
0		37.906		
0		55.388		
0		84.438		
0		67.536		
0		100.814		
0		36.413		
0		11.877		
0				
0		13.812		
0		18.104		
0		21.206		
0				
0		94.024		
0		74.804		
0		105.398		
0		79.829		
0		61.777		
0				
0		101.098		
0		142.963		
0		37.436		
0		131.756		
0		31.384		
0				
0		24.275		
0				
0				
0		84.166		
0		8.718		
0		23.889		
0		15.754		
0		23.288		
0		25.150		
0		52.532		
0		47.243		
0		24.041		
0		57.784		
0		58.785		
0		67.537		

0		70.968		
0		92.048		
0		152.131		
0		68.184		
0		41.804		
0		61.914		
0		87.394		
0		51.206		
0				
0		54.433		
0		31.923		
0		37.665		
0		39.457		
0		33.412		
0		33.331		
0		29.127		
0		31.824		
0		43.752		
0		32.816		
0		28.858		
0		115.126		
0		36.027		
0		31.921		
0				
0				
0		23.406		
0		59.655		
0		57.775		
0		50.147		
0		115.584		
0		32.723		
0		62.622		
0		61.629		
0			10.691	11.213
0			18.210	43.061
0			19.867	11.174
0			39.220	19.216
0			18.168	12.247
0			6.191	
0			39.465	135.841
0			80.774	107.908
0			79.155	109.159
0			54.462	
0			42.776	38.596
0			57.544	
0			44.678	
0				47.434
0				41.733
0				28.231
0				37.760

0				
0				
0				
0				
0				
0				
0				
0				
0				
0				
0				
2			42.323	38.169
2			41.978	35.201
2			53.147	38.688
1			38.845	
1				
1				
1				
2			33.702	26.655
6				46.704
1			58.144	134.116
1				33.377
1				
				131.689
			52.691	
				95.226
			21.284	28.423
			29.744	
				32.648
				118.626
1				
2				
2				
2				
2				
5				
5				
5				
3			38.571	21.323
3			37.507	18.578
3			42.021	26.416
3			31.411	43.803
3				

2				67.555
4				
3				
		100.426		
		47.768		
		16.726		
		49.662		
		68.790		
		82.081		
4				
4				
4			45.521	21.997
4			68.200	55.250
4				34.772
4			40.516	28.376
4			27.750	96.593
4			77.892	
4			59.500	
4			29.091	
4		40.169	49.438	
4		28.278	32.310	18.852
4		60.047	31.474	21.724
4		43.184	36.485	
4				
4		47.805	51.818	
4			56.524	
4				
4			19.312	12.352
4	1	42.648	24, 579	35.525
4	1	19.131	24.207	6.107
4	1	28.896	24.881	20.288
4	1	32.113		
4	1	24.440	20.494	22.831
4	0	24.577	11.875	9.672
4	1	17.907	23.641	13.651
4	1	30.546	24.182	25.976
4	1	34.866	36.648	20.108
4	1	48.204	15.609	15.953
4	1		48.192	15.527
4	1	14.809	12.546	15.768
4	0	31.227	21.046	
4	1	48.472	43.757	
4	0	29.692	18.010	11.878
4	1	56.325	35.349	33.062
4	1		33.006	
4	1	25.355		32.522
4	1			26.080
4	0	61.662	58.861	
4	0	40.932	39, 512	

4	0	33.067	24.826	
4	0	35.386		
4	0	50.859		
4	1	9.977		
4	1	24.446	36.595	
4	1	15.338	23.129	
4	0			
4	1			
4	0			

mTOR_P_Nor	mTOR_P_Peri	mTOR_P_Tum	LC3_R_Nor	LC3_R_Peri
			91.278	
			87.343	
			114.639	
			116.944	
			36.564	
			126.163	
			64.420	
			60.823	
			72.314	
			129.600	
			121.233	
			120.503	
			100.313	
			78.358	
			85.492	
			48.070	
			140.787	
			90.106	
			123.608	
			96.219	
			106.301	
			85.889	
			107.675	
			153.836	
			106.932	
			186.486	
			161.108	
			74.157	
			75.286	
			58.506	
			75.234	
			71.886	
			105.433	
			107.040	
			101.898	
			208.794	
			103.916	
			66.988	
			124.496	
			120.850	

			135.194	
			165.229	
			179.894	
			132.900	
			81.254	
			95.495	
			104.909	
			124.576	
			106.993	
			44.442	
			110.112	
			89.982	
			94.067	
			73.071	
			36.181	
			54.163	
			33.819	
			46.201	
			61.668	
			119.080	
			59.488	
			74.315	
			70.394	
			122.242	
			75.570	
			64.979	
			143.570	
			51.520	
			119.203	
			87.013	
				15.841
				51.838
				40.870
				39.943
				23.088
				21.686
				46.635
	0.3278	0.0471		105.070
				54.200
				51.148
	0.1188	0.1300		83.601
	0.1075	0.8092		67.761
	0.0955	0.1764		55.764
		0.2960		
	0.3190	0.1052		
	0.2049	0.1054		
		0.1346		

	0.1129			
	0.3278	0.0471		
	3.1224	2.5451		
	0.2480	0.1431		
	0.4310	0.7253		
	0.7573			
	0.5193	0.7061		
	0.1257	0.3429		
				70.634
				69.599
				69.427
				49.791
				116.225
	0.2899	0.1832		57.514
	0.7076			
	0.6483	0.6694		63.056
	0.0761	0.0297		
	0.6514	0.1066		21.403
	0.2681	0.7853		39.419
	0.1085	0.0445		
	0.0412	0.0368		
	0.0865	0.0481		
	1.5454	0.6281		
	0.0678	0.0324		
	0.2039			
	0.5769	0.6857		
	0.6457	0.2060		
	1.3288	0.4988		
	0.0608			
				86.748
				35.049
				72.047
				48.139

	0.3083			
			195.563	
			100.935	
			21.678	
			70.436	
			58.136	
			117.309	
			84.162	
	0.2007			41.594
	0.1979			50.724
				64.914
	0.3445	0.4245		30.831
	0.2430			86.821
	0.1038			53.170
	0.1153	0.1091		
0.6590	0.1579	0.0899	51.896	99.406
0.0328	0.0381	0.3009	54.855	71.287
0.0709	0.0515	0.4827	91.276	58.252
			36.551	49.951
0.0801	0.2558	0.7566		
0.1033	0.1263	0.3432	46.818	
0.2189	0.5129	0.5170		99.570
0.3290	0.6756	1.0626		
0.7734	0.5077	0.1526		15.961
0.0771	0.1385	0.2823	80.287	29.717
0.3364	0.5436	0.4289	47.188	58.789
0.2777	0.1825	0.4044	89.269	61.164
0.1278	0.1199	0.3973	118.283	
0.3048	0.3174	0.3415	102.847	64.582
0.8283	0.7472	0.4713	67.150	20.541
0.1902	0.2575	0.3899	47.761	83.132
1.1424	0.2861	0.2440	88.521	104.456
0.0379	0.0795		100.308	99.260
0.4019	0.4623	0.1563	99.938	47.201
0.0606	0.2437	0.0727		114.301
0.3409	0.3987	0.3611	32.640	43.074
0.2011	0.8234	0.7296	92.589	58.063
0.1138	0.4315	0.6961	146.617	159.829
0.3514	0.5116	0.8164	92.620	71.862
0.6461	0.7055	0.7006	135.959	69.704
1.0411	1.1914	0.8199		156.633
0.1955	0.2786	0.4165	63.165	
0.1652	0.1295	0.1873		
0.3360	0.1330	1.0434	65.410	57.337
0.3232	0.3367	0.9034	53.449	35.082

0.8112	1.0894	1.0442	103.449	37.728
0.9026	1.1355	0.7412	49.462	
0.1024	0.6295	0.9843	71.314	
0.6472		0.7921	19.990	
0.4826	0.1577	0.4828	40.508	49.476
0.3329	0.0505	0.4233	22.191	36.568
0.5249	0.5704	0.527		
0.3822	0.2755	0.4100		
0.1002	0.4773	0.3909		

LC3_R_Tum	LC3_P_Nor	LC3_P_Peri	LC3_P_Tum	p62 Prot nor

12.623				
22.139				
8.415				
25.705				
12.818				
13.601				
48.952		0.0145	0.1798	
140.230		0.1624	0.1481	
		0.1867		
64.214		0.1826	0.5650	
		0.3093	0.2294	
		0.0799		
51.557			0.0940	
34.002		0.3675	1.2433	
29.667		0.4150	0.1225	
10.875				

		0.1336	0.2026	
		0.0979	0.092	
		0.0106	0.1243	
		0.4492	1.1044	
		0.0808	0.2263	
		0.1824	0.5352	
		0.1086		
		0.0955	0.0873	
		0.1001	0.2027	
28.386				
47.032				
72.630				
53.153				
39.526				
42.254		0.1383	0.0711	
15.629				
		0.2427		
		0.3783	0.2920	
21.650		0.9650	1.9795	
19.389		0.6721	0.0725	
		0.2506	0.0672	
19.800		0.3903	0.7109	
		0.0537	0.7942	
		0.2742	0.3174	
65.245		0.1350	0.0695	
		0.0137	0.0956	
		0.1985		
		0.0981	0.0762	
		0.4038	0.5144	
		0.1163		
39.572				
13.395				
24.219				
19.493				

		0.2270		
8.126		0.6253		
11.437		1.1819		
6.317				
17.868				
		1.1842	0.1146	
		0.4431		
		0.8876		
		0.4977	0.2615	
	0.3872	0.2152	0.1441	
31.095	0.4099	0.4749	0.6519	1.2957
37.093	0.3347	0.4092	1.0848	
	0.7364	0.6370	0.7060	0.0912
	1.1471	0.7939	0.4906	0.4356
	0.1540	0.4651	2.4376	
	0.3505	0.4778	1.2947	0.2135
	0.1455	0.2245	0.7635	0.1196
5.493	0.5447	0.7681	5.1459	0.5194
45.839	0.9783	1.0152	5.3811	0.1824
27.207	0.98	0.9648		0.3371
5.447	0.3634	0.3621	0.4557	0.0996
	0.3945	0.1880	0.5167	0.2062
37.623	1.1068	0.3258	0.2642	0.4023
14.685	0.1435	0.3231	0.2676	2.0316
25.741	0.0007	0.5285	0.9352	0.4245
53.679	0.2157	0.3327	0.4217	0.2142
45.414	0.5735	0.5871	0.9927	0.4547
46.759	0.4369	0.5531	0.4417	1.5637
26.323	1.2138	1.4782	3.7280	0.1264
30.071	0.3856	0.4398	0.9225	0.3945
	0.3296	0.2216	0.5645	0.3561
	0.6513	0.9304	1.5187	0.3886
39.355	0.2216	0.1709	0.4980	0.1844
48.109	0.4673	0.5830	0.3239	0.3390
	0.2884	0.2192	0.2459	0.4011
32.772	0.1759	0.2241	0.3164	0.3113
18.521	0.2715	0.2368	0.2380	0.1440
	0.3706	0.1647	0.2484	0.2550
	0.3118	0.5847	0.6384	0.4266

	0.2339	0.1408	0.9342	1.1042
	0.0467	0.0638	0.3348	2.4729
	0.9304	0.4602	0.4256	0.4285
	0.4373	0.4486	0.4474	0.1675
	0.7195	0.7245	1.1244	0.5683
	0.1233	0.2308	2.4806	2.4975
	0.1259	0.1914	0.5925	0.5272
	0.1631	0.2414	0.1265	0.3024
	0.1886	0.2903	0.2388	0.3047

p62 Prot per	p62 Prot tum	ULK7_P_nor	ULK_P_peri	ULK_P_tum

0.5610	0.225			
0.1443	0.1797			
0.1728				
0.3824	0.2156			
0.3430	2.2973		0.9298	
0.0834	3.8020		0.3802	0.3354
	3.651		0.3325	0.2007
0.7016				
0.2360	0.4270			0.3276
	3.108		0.5707	0.7531

			0.4781	0.6175
0.1719	0.1164			0.8351
0.0621	1.436			
0.1881	1.3472			
0.2884	1.2346			
0.1399	3.9569		0.6964	0.6607
0.0918				
0.597	0.4996			
			0.7555	
0.0984	1.480			
0.0797	0.2971			
			0.9227	0.6355
			0.3907	
0.7259	1.7436			
0.4330	0.2970			
0.3041	0.7078			
0.5777			0.643	0.7053
0.3176	0.7203		0.7059	0.6595
0.1343	1.3586		0.4006	0.7166
0.2024	0.4535		0.4359	0.6187
0.1067	2.5161			
0.2953			0.2845	0.5817
			0.4533	
			1.3049	0.7581
0.1092	0.6455			
0.0981	0.6354			
0.2658	0.0859		0.9227	1,048
0.3036			0.957	

0.0498				
			0.1684	
0.3628				
0.2071				
0.1717				
0.6664				
0.4813			0.4822	
			0.7809	
0.1722				
0.0829				
0.6949				
0.0774	0.1288			
			0.5074	
0.4530	0.1249		0.6755	0.4788
		1.1379	1.2174	0.8959
0.1381	0.2303	1.1361	0.7962	0.7498
0.1911	0.3099	0.9474		0.4879
0.1605	0.2321	1.1868	1.1601	0.6976
0.5527	0.3578	0.6789	0.6793	0.217
0.7927	1.8225			
0.2229	0.5779			
0.2519	0.1561	0.7383	0.8039	0.8669
0.1411	0.2083	0.1872	0.1154	0.0518
0.1979	0.3678	0.276	0.2379	0.0961
0.2436	1.0152	0.3698	0.3584	0.3137
0.7901	0.9752	0.6692	0.6995	0.2466
0.4481	0.4112			
0.1804	0.1062			
0.5324	0.4686			
0.4251	0.258			
0.1636	0.2250	0.1149	0.1145	0.0724
0.2800	0.7858	0.0482	0.1085	0.0376
0.3545	0.6062	0.1419	0.4399	0.1066
0.2836	0.1557	0.1168	0.0502	0.0582
0.1946	0.5276			
0.1807	0.1189	0.4258	0.5159	0.3264
0.2155	0.3481			
0.2455	0.3445	0.2852	0.1877	0.1936
0.1795	0.3334	0.1194	0.1603	0.1121
0.2601	0.3895	0.1787	0.1479	0.1696
0.2547	0.1102	0.3775	0.535	0.1365

2.0964	1.7498			
1.3250	1.0887	0.4711	0.6254	0.2652
0.1670	0.8603			
0.3047	0.3592			
0.3957	0.3183			
0.9962	0.2689			
0.2764	0.2266			
0.2956	0.4289	0.2576	0.248	0.0373
0.3456	0.7928	0.1574	0.2206	0.1313
		0.3344	0.6304	0.5341
		0.3337	0.3161	0.0785

RAPTOR_P_nor	RAPTOR_P_peri	RAPTOR_P_tum		

	0.4419	0.1423		
	0.1643	0.1404		
	0.3335			
	0.1674	0.1048		
	0.2905	0.2806		
	0.0835	0.2132		
		0.2801		
	0.2824	0.2491		

	0.2066	0.8554		
		0.4796		
	0.2861	0.7844		
	0.4755	0.1216		
	0.1138	0.0781		
	0.1286			
	0.1981	0.18		
	0.122	0.066		
	0.27			
	0.0996	0.2237		
	0.4244	0.2044		
	0.321	0.1774		
	0.5641			
	0.7292			
	0.19	0.8939		
	1.1699	0.7977		
	1.0751	0.9908		
	0.9367	0.7482		
	1.1515	0.965		
	0.3972	0.6086		
	0.4492	0.439		
	0.1456	0.0596		
	0.181			
	0.0613	0.0724		
	0.4213	0.2156		
	0.121	0.1446		
	0.6281	0.5976		
	0.4205			

	0.1227			
	0.5871			
	0.5438			
	0.2982			
	0.3604			
	0.4423			
	0.2491	0.2208		
	0.3577			
	0.9581			
	0.1809	0.3438		
0.5248	0.2982	0.2581		
0.2088	0.1543	0.1538		
0.1811	0.1716	0.7606		
0.2319	0.1269	0.1041		
0.231	0.2556	1.1454		
0.2289	0.1474	0.1647		
0.2051	0.2343	0.695		
0.5826	0.7296	0.7087		
0.1336	0.1265	0.109		
0.2232	0.1957	0.1247		
0.1523	0.1167	0.1298		
0.4435	0.402	0.3531		
0.4331	0.8787	0.2546		
0.1367	0.1128	0.1511		
0.8507	0.9082			
0.7447	0.6963	0.7658		
0.1317	0.2545	0.4389		
0.892	1.0634	1.3440		
0.5053	0.586	1.169		
0.5997	0.5929	0.9411		
1.0132	0.7436	1.4924		
0.7054	0.6446			
1.1185	1.0629	1.1324		
0.5614	0.7181	0.5487		
0.2384	0.1752	0.1429		

0.5791	0.4894	1.1162		
0.1692	0.1409	0.2345		
0.3066	0.4601	0.3627		
0.4448	0.5139	0.33		
0.5045	0.3312	0.3081		
0.1072		0.2393		
0.3594	0.5519	0.4357		
0.8191	0.7535	0.6835		
0.8864	1.0426	0.6392		
1.5244	1.3710	1.9939		
0.8807	1.5242	2.3791		
