# Supplementary material for: Gene and protein expression of mTOR and LC3 in hepatocellular carcinoma, colorectal liver metastasis and “normal” liver tissues
Source: PLoS One. 2020 Dec 23;15(12):e0244356. doi: 10.1371/journal.pone.0244356 (PMC7757890; doi:10.1371/journal.pone.0244356)
Supplement: Attachment — Submitted filename: Response to Reviewers.docx [file pone.0244356.s004.docx]

**Response to Reviewers**

Dear Editor,

we thank the Academic Editor and the Reviewers for the careful attention and the comments given to our manuscript PONE-D-20-07836 “Gene and protein expression of mTOR and LC3 in hepatocellular carcinoma, colorectal liver metastasis  and "normal" liver tissues”. We thoroughly followed their suggestions and we feel that the manuscript ultimately improved. We hope that the present revised version of our manuscript fully meets the criteria of Plos One and that will be considered for publication.

Here a point by point answer to Academic Editor and the Reviewer #2

**Academic Editor:** To make valid conclusions about the autophagy status, the authors should assess the levels of selective autophagy targets such as SQSTM1/p62 or NBR1, as well as the activation (phosphorylation) of key autophagy signaling molecules such as ULK1.

Answer: We thank the Academic Editor for the comments and for giving us the opportunity to resubmit a revised version of the manuscript. We agree with the suggestion, therefore, we assessed the protein expression of p62, ULK1 and Raptor in the studied tissues. We added the corresponding paragraphs in the results session and in the discussion section. All the inserted new paragraphs are written in blue.

Also, as the p values have already been shown in the figures, there is no need to repeat them in the text, it makes the manuscript difficult to follow.

Answer: done as requested. We showed the q-values in the text and deleted the P-values.

**Reviewer 2**

This is a well performed study showing the levels of mTOR and LC3 in hepatocellular carcinoma, colorectal liver metastasis and "normal" liver tissues. Previous works show the important role that autophagy can have in liver diseases. In this study, the authors show the different levels of protein and mRNA expression in mTOR and LC3 that primary and secondary hepatic tumors present. I have no substantial objections to the experimental work, which is straightforward and presented in a clear manner. As such, these data support the conclusions.

We thank Reviewer 2 for the positive comments.

However, for completeness, there are a number of points that should be addressed by the authors.

Minor points:
•     In Table 1 (Etiological characteristics of subject), in the HCC column it indicates that the number of patients is 57 but when separated by gender they add up to 56 (20 female and 36 male)

Answer: done as requested. We corrected the total number.

•     In line 268 of the document, the paragraph talks about Figure 1D but indicates Figure 1C

Answer: done as requested.

•     In the document I would add some information that helps your understanding:
-     In line 282 of the document the CIRR value is not indicated

Answer: done as requested. We apologize for the oversight.

-     In line 305 of the document the value of M is not indicated

Answer: done as requested. We apologize for the oversight.

-     At the end of the paragraph that begins to describe figure 2B I would add Fig 2B, on line 301.

Answer: done as requested.

•     In Figure 1A, we can see the difference in the errors of CH and CIRR, but in the document on line 254 it indicates that CH (53.3±30), CIRR (56.2±30). check

Answer: done as requested. Figure 1 was redone.

•     In Figure 4 A and B, we can see that the PHCC and HCC values are close to 0.2, but in line 320 of the document it indicates values of  (0.4±0.5) or (0.39±0.5) respectively. Check

Answer: done as requested. Figure 4 was redone.

•     In Table 2 there are two questions that I would like to clarify:
-     First in the mRNA correlation column of mTOR and LC3 in CH HBV + there is no coefficient value but it does have p value.

Answer: done as requested. We apologize for the oversight.

-     Second, I do not understand the meaning of negative values greater than 1, such as the NM NA value of the same column as before. Spearman's correlation coefficient values are between 1 and -1.

Answer: done as requested. We apologize for the typos, all the values greater than 1 where missing the “0,” above.

•     As the authors explain that the M samples present the lowest levels of mRNA of all the samples in the mTOR and LC3 gene, but then they observe the highest expression values of both proteins in M.

Answer: Indeed, as observed for mTOR and LC3 gene expression in M, the protein expression of mTOR and LC3 was also decreased if we considered the ratio between mTOR /beta-actin and between LC3-I/beta-actin. The increase found is due to the ratio between the mTOR phosphorylated and non-phosphorylated form and between LC3-II and LC3-I form.

•     Figure 3 presents 9 representative samples of all the samples studied that are included in the densitometers of figure 4. I believe that the films Western blots of all tumors can be displayed on platforms such as (Mendeley Data) for this to be consulted. (Justification Point 3)

Answer: we will follow this suggestion.

•     The discussion talks about autophagy (line 519 of the document), but the document does not provide enough data to determine the state of autophagy, in my opinion it is difficult to determine the state of autophagy based on two genes or proteins only. It is more convenient to indicate the possible activation of autophagy, or something similar.

Answer: As suggested by the Academic Editor we assessed the protein expression of p62, ULK1 and Raptor in the studied tissues. We added the corresponding paragraphs in the results session and in the discussion section. All the inserted new paragraphs are written in blue.

•     Finally, the quality of the images of all the figures is very low, which makes it difficult to understand the text. I think that they should be replaced by higher quality images that allow greater visibility.

Answer: done as requested.

==============================

**Journal Requirements:**

2.We noticed minor instances of text overlap with the following previous publication(s), which need to be addressed:

The text that needs to be addressed involves the Introduction section (lines 93-96)

Answer: done as requested. We rephrased the sentence.

3. Please provide additional details regarding participant consent. In the ethics statement in the Methods and online submission information, please ensure that you have specified what type of consent you obtained (for instance, written or verbal, and if verbal, how it was documented and witnessed).

Answer: done as requested. See Methods section

4. In your Methods section, please provide additional information about the participant recruitment method and the demographic details of your participants. Please ensure you have provided sufficient details to replicate the analyses such as: a) the recruitment date range (month and year), b) a description of any inclusion/exclusion criteria that were applied to participant recruitment, c) a description of how participants were recruited.

 Answer: done as requested. See Methods section, “Patients and characteristics” paragraph

5. Please provide a sample size and power calculation in the Methods, or discuss the reasons for not performing one before study initiation.

 Answer: done as requested. See Methods section, “Statistical analysis” paragraph and supporting information, supplementary table 1.

**Additional PLOS ONE requirements (Oct 05 2020 04:59AM)**

1) Please note that PLOS ONE requires submissions reporting blot or gel data to comply in full with the reporting requirements described at https://journals.plos.org/plosone/s/figures#loc-blot-and-gel-reporting-requirements. We now require authors to provide the original unadjusted and uncropped images for any blot or gel data reported in PLOS ONE submissions. In our internal checks for your submission, we noted that you did not provide original raw image files supporting blot/gel data in response to our previous request.

 Answer: done as requested. See Mendeley repository: <https://data.mendeley.com/datasets/kg2rvwnprh/draft?a=eca9af40-018a-4200-9176-50a889bf5898>

2) Please include a separate legend for each figure within your manuscript.

 Answer: done as requested. Legends are at the end of the manuscript, after the references section.
